# Intrapericardial Fibrinolytic Therapy in Purulent Pericarditis: A Review of Two Cases

**DOI:** 10.7759/cureus.35172

**Published:** 2023-02-19

**Authors:** Abhushan Poudyal, Ajoe J Kattoor, Anoj Shahi, Nataliya Pyslar, Ray Sawaqed

**Affiliations:** 1 Cardiology, John H. Stroger, Jr. Hospital of Cook County, Chicago, USA; 2 Internal Medicine, John H. Stroger, Jr. Hospital of Cook County, Chicago, USA; 3 Cardiothoracic Surgery, John H. Stroger, Jr. Hospital of Cook County, Chicago, USA

**Keywords:** pericardiectomy, constrictive pericarditis, tissue plasminogen activator, pericardial space, purulent pericarditis

## Abstract

Purulent pericarditis is the infection of the pericardial space with pus formation. High mortality and morbidity can be explained by cardiac tamponade and septic shock in the acute phase, while chronically, it can lead to recurrent purulent pericarditis and constrictive pericarditis. We present two cases of purulent pericarditis treated with intrapericardial recombinant tissue plasminogen activator (r-tPA) for three consecutive days in addition to surgical pericardial drainage. In both instances, loculated effusions and re-accumulation of pericardial fluid persisted despite adequate antibiotic coverage and surgical drainage. Intrapericardial fibrinolysis was considered a less invasive alternative to extensive surgery to prevent constrictive pericarditis and improve clinical outcomes. Both patients had complete clinical recovery and there was no evidence of constrictive pericarditis during follow-up. There is scant literature regarding r-tPA therapy for purulent pericarditis, most of which is limited to case reports or case series. The most commonly used regimen is three doses of tPA administered into the pericardial space over three days. It is a safe and potentially effective therapy in preventing constrictive pericarditis and need of pericardiectomy.

## Introduction

Purulent pericarditis is characterized by pus in the pericardial space from an infective process. There are no established definitions for purulent pericarditis. In the study by Rubin et al., patients fulfilling at least two of the following three criteria were considered to have purulent pericarditis: demonstration of gross or microscopic purulence (more than 30,000 WBC per mm^3^ with more than 90% polymorphonuclear leukocytes) in pericardial fluid, demonstration of bacteria or fungi on Gram stain of pericardial fluid, and isolation of a bacterium (other than *Mycobacterium tuberculosis*) or fungus from cultures of pericardial tissue or fluid, or from cultures of immediately contiguous structures and/or blood [1]. Previously reported mortality rate for purulent pericarditis is between 20% and 30%, however, a much lower in-patient mortality rate (2.3%) has been reported in a new study [2,3]. Patients who recover are at risk for constrictive pericarditis due to adhesions and scarring [2]. Fibrin plays a major role in persistent purulent pericarditis, risking progression to constriction. Fibrinolytic agents, including r-tPA, can potentially prevent this complication by inducing fibrinolysis via plasminogen activation. We present two cases of purulent pericarditis treated with intrapericardial r-tPA and review the current evidence regarding the use of fibrinolysis in purulent pericarditis.

## Case presentation

Case 1

**Figure 1:**
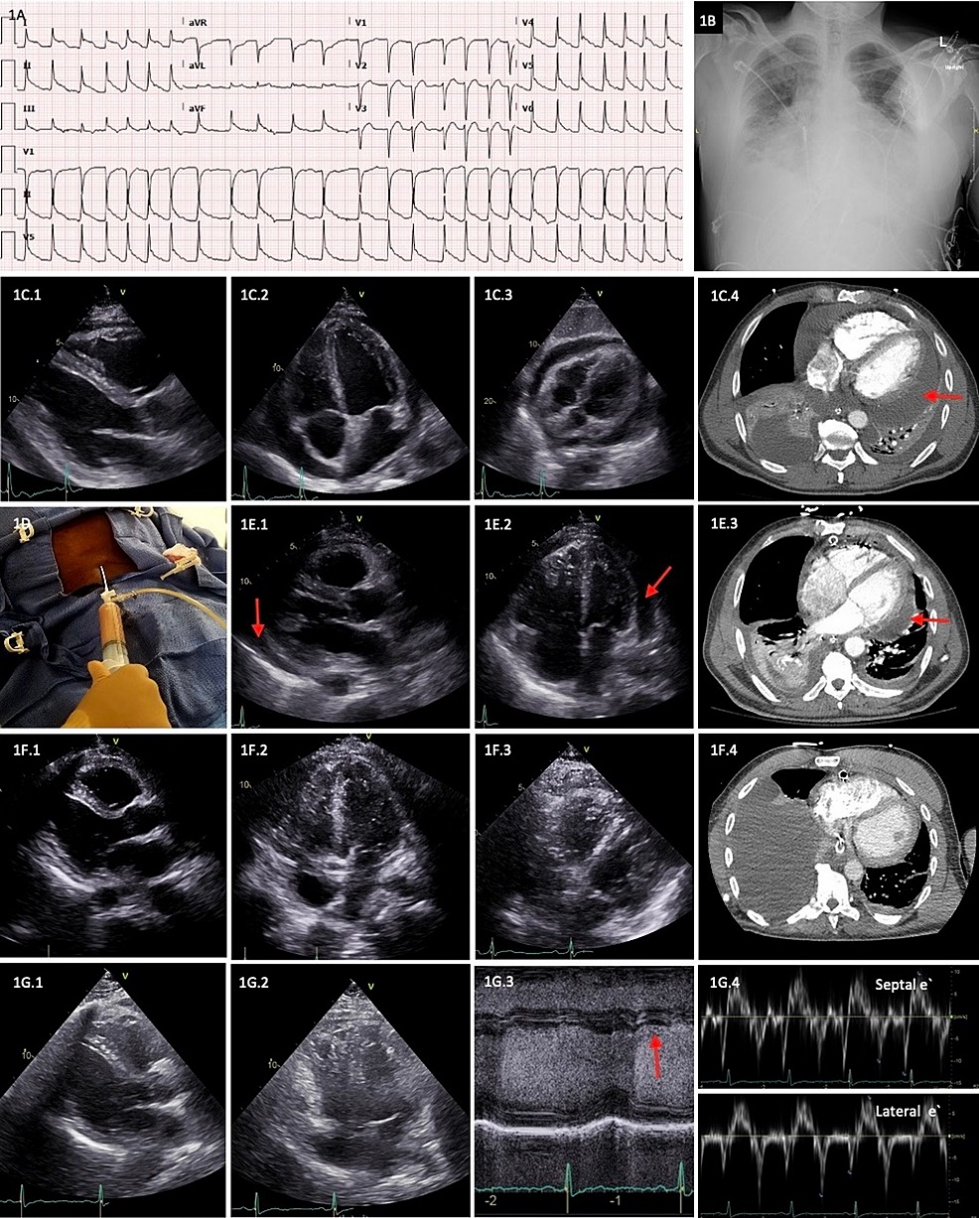
ECG, chest x-ray, echocardiogram, and CT of case 1. (A) ECG showing atrial fibrillation and diffuse ST elevations consistent with pericarditis; (B) chest x-ray demonstrating cardiomegaly, bilateral interstitial opacities predominantly in the lower lung fields and blunting of costophrenic angles suggestive of pulmonary edema and pleural effusion; (C) day 1 - transthoracic echocardiogram and CT scan demonstrating circumferential pericardial effusion (red arrow; C.1: parasternal long axis, C.2: apical 4 chamber, C.3: subcostal views, and C.4: axial CT image); (D) day 2 - pericardiocentesis draining purulent fluid; (E) day 4 - transthoracic echocardiogram and CT scan (day 2 after surgical pericardial drain placement) showing residual loculated pericardial effusion (red arrow; E.1: parasternal long axis, E.2: apical 4 chamber views, and E.3: axial CT image); (F) day 8 - transthoracic echocardiogram and CT after five days of intrapericardial r-tPA administration showing near complete resolution of the effusion (F.1: parasternal long axis, F.2: apical 4 chamber, F.3: subcostal views, and F.4: axial CT image); (G) 2-year follow-up - transthoracic echocardiogram demonstrating absence of pericardial effusion and constrictive physiology (G.1: parasternal long axis and G.2: apical 4 chamber views, G.3: M mode demonstrating absence of early-mid diastolic notching {red arrow}, and G.4: absence of annulus reversus in the e` septal and e` lateral).

A 53-year-old man was brought to the emergency room for altered mentation. He was in acute hypoxic and hypercapnic respiratory failure with unstable vital signs (blood pressure 75/54 mmHg, heart rate 148 beats per minute (bpm), respiratory rate 8 breaths/min, and SpO_2_ 68% on room air). He had been treated for pneumonia at an outside hospital four days back and was discharged on antibiotics. The patient was an active tobacco, heroin, and cocaine abuser. He received naloxone without any significant response. Baseline laboratory values are shown in Table 1. Electrocardiogram (ECG) revealed atrial fibrillation with rapid ventricular rate and diffuse ST-segment elevation suggestive of acute pericarditis (Figure 1A). Chest x-ray showed cardiomegaly with cephalization of the pulmonary vasculature, bilateral interstitial opacities, and blunting of costophrenic angles (Figure 1B). Emergent intubation was performed for respiratory failure and altered mentation. Intravenous fluids, vancomycin, and cefepime were started for septic shock and vasopressor support for persistent hypotension. On day 1, the transthoracic echocardiogram (TTE) demonstrated moderate diffuse hypokinesis with left ventricular ejection fraction of 35% and a large, free-flowing pericardial effusion without features of cardiac tamponade (Figure 1C). Right heart catheterization excluded tamponade physiology but revealed depressed cardiac output (Table 2). In the next 24 hours, his vasopressor requirement increased. A repeat TTE showed pericardial effusion expansion with mildly fibrinous appearance and evidence of exaggerated ventricular interdependence. Urgent pericardiocentesis was performed on day two, draining 720 mL of grossly purulent fluid (Figure 1D and Table 3). There were pockets of pericardial fluid despite the insertion of percutaneous pericardial drain. Emergent surgical washout was performed on the same day, draining an additional 300 mL of purulent pericardial fluid, and two pericardial drains were placed. Despite surgical drainage, moderate loculated pericardial effusion persisted (Figure 1E). Pericardiectomy was offered but the patient declined. Therefore, to facilitate complete pericardial drainage of purulent material r-tPA (alteplase) was infused into the pericardial space (10 mg daily for three consecutive days), beginning on day five. Patient’s hemodynamics gradually improved, and he was extubated on day six. Despite significant improvement in the pericardial effusion size on day eight, there were new findings of enhanced interventricular interaction and exaggerated respirophasic transmitral and tricuspid doppler velocity variation with expiratory diastolic hepatic venous doppler reversal, suggestive of constrictive pericarditis (Figure 1F). Gram staining of pericardial fluid showed Gram-positive cocci, but cultures were sterile. Infection with a nutritionally deficient Streptococcus was presumed. Each pericardial drain was removed on the 14th and 15th day of admission. Patient was treated with intravenous ampicillin-sulbactam for four weeks after source control, followed by oral amoxicillin-clavulanate for an additional two weeks. Colchicine was prescribed for three months to prevent recurrent pericarditis. He was readmitted four months later for community-acquired pneumonia and atrial fibrillation with rapid ventricular rate. TTE revealed a trivial pericardial effusion with no features of constrictive pericarditis. Follow-up TTEs were obtained at 19 months and 24 months from the initial admission and demonstrated mildly elevated pulmonary artery pressure and calcifications in the pericardial space but no evidence of constrictive physiology (Figure 1G). However, the patient continued to have mild dyspnea on exertion.

**Table 1:** Baseline laboratory data. BUN: blood urea nitrogen; ALP: alkaline phosphatase; GGT: gamma-glutamyl transferase; AST: aspartate transaminase; ALT: alanine transaminase; LDH: lactate dehydrogenase; BNP: brain natriuretic peptide

Variable	Case 1	Case 2	Reference range
Sodium (mEq/L)	128	135	135-145
Potassium (mEq/L)	4.9	3.8	3.5-5
Chloride (mEq/L)	91	95	100-110
Bicarbonate (mEq/L)	24	26	23-31
BUN (mg/dL)	64	65	9-20
Creatinine (mg/dL)	3.5	5.3	0.6-1.4
Glucose (mg/dL)	75	179	65-110
Calcium (mg/dL)	8.1	8.5	8.5-10.5
Total protein (g/dL)	5.7	7.3	6.4-8.3
Albumin (g/dL)	3.1	2.7	3.8-5.2
Total bilirubin (mg/dL)	0.5	2.1	0.2-1.2
ALP (U/L)	42	102	20-120
GGT (U/L)	9	18	3-60
AST (U/L)	54	87	0-40
ALT (U/L)	17	56	5-35
LDH (U/L)	269	610	85-210
Hemoglobin (g/dL)	14.1	14.1	12.9-16.8
White blood cells (K/uL)	13.8	23.7	4.4-10.6
Differential leukocyte count			
1. Neutrophil (%)	36	70	45.3-74.5
2. Bands (%)	55	6	-
3. Lymphocyte	3	6.9	18.1-43.2
4. Monocyte	1	2	4-11.1
Platelets (K/uL)	233	434	161-369
Troponin-I (ng/mL)	14.1	0.044	0.000-0.039
BNP (pg/mL)	1315	-	<100
Venous blood gas lactate (mmol/L)	3.80	6.60	0.50-1.60

**Table 2:** Right heart catheterization data for case 1. RA: right atrium; RV: right ventricle; PA: pulmonary artery; PCWP: pulmonary capillary wedge pressure

Variable	Value
RA pressure (mmHg)	14
RV pressure - systolic/end-diastolic (mmHg)	41/7
PA pressure - systolic/diastolic/mean (mmHg)	42/19/27
PCWP (mmHg)	20
PA oxygen saturation (%)	57
Systemic oxygen saturation (%)	99
Cardiac output (L/min)	4
Cardiac index (L/min/m^2^)	1.9

**Table 3:** Pericardial fluid analysis.

Variable	Case 1	Case 2
Color	Pink	Pale yellow
Appearance	Cloudy	Turbid
Red blood cells (/uL)	1500	1830
White blood cells (/uL)	33,500	56,000
1. Polymorphonuclear cells (%)	96	96
2. Mononuclear cells (%)	4	2
Amylase (U/L)	15	-
Protein (g/dL)	4.8	-
Albumin (g/dL)	2.8	-
Glucose (mg/dL)	<10	-
Lactate dehydrogenase (U/L)	10,148	-

Case 2

**Figure 2:**
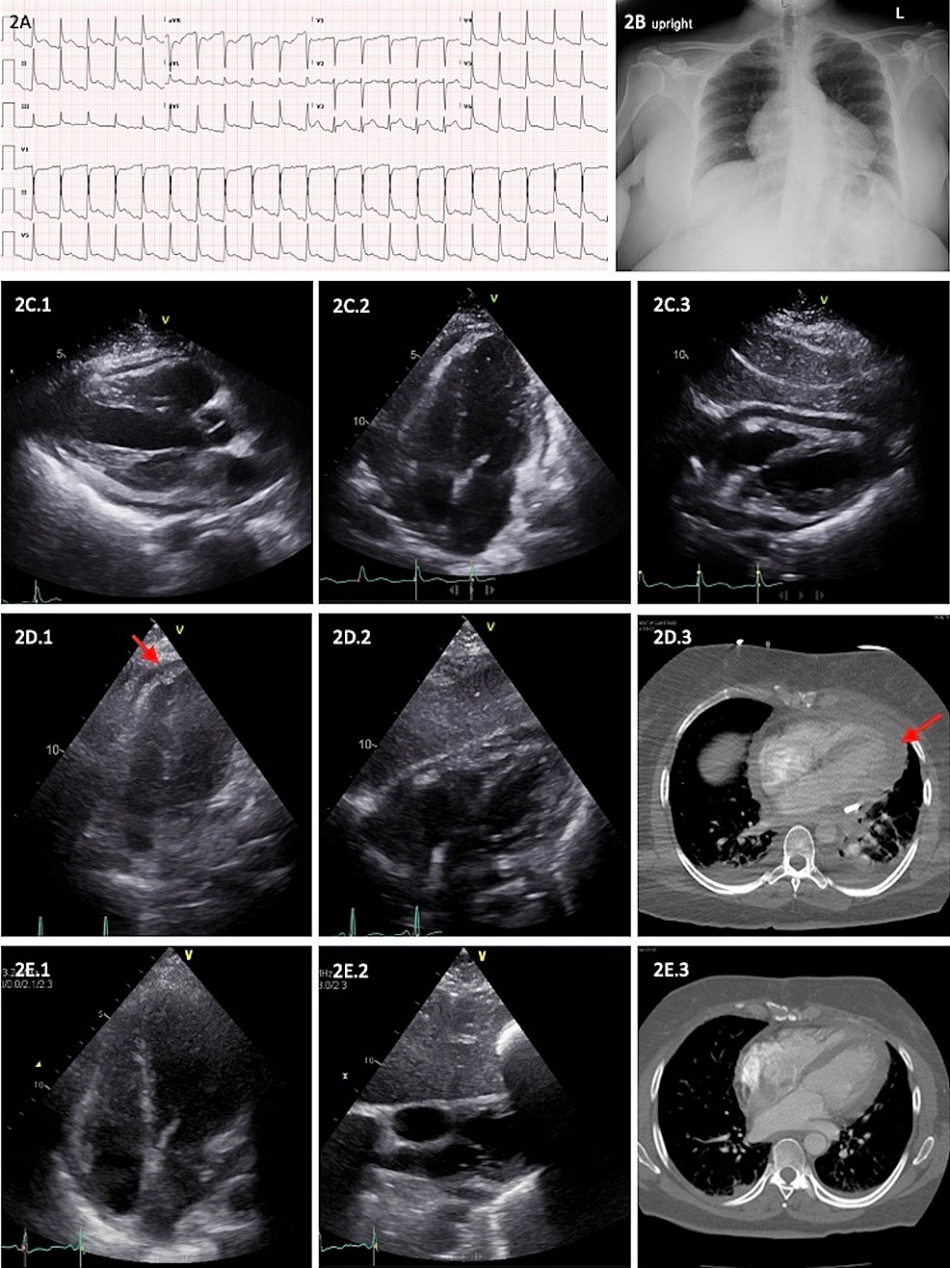
ECG, chest x-ray, echocardiogram, and CT of case 2. (A) ECG showing atrial fibrillation and diffuse ST elevations consistent with pericarditis; (B) chest x-ray demonstrating cardiomegaly; (C) day 1 - transthoracic echocardiogram demonstrating moderate circumferential pericardial effusion (C.1: parasternal long axis, C.2: apical 4 chamber, and C.3: subcostal views); (D) day 6 - transthoracic echocardiogram and CT scan (day 3 after surgical pericardial drain placement) showing residual loculated pericardial effusion near the LV apex (red arrow, D.1: apical 4 chamber, D.2: subcostal views, and D.3: axial CT image); (E) post-intrapericardial r-tPA transthoracic echocardiogram on day 23 and CT chest on day 17, showing near complete resolution of the effusion (E.1: apical 4 chamber, E.2: subcostal views, and E.3: axial CT image).

A 52-year-old woman presented with cough, fever, and myalgia to the emergency department. She was hypotensive and tachycardic on arrival (heart rate 135 bpm, blood pressure 90/51 mmHg, respiratory rate 21 breaths/min, and SpO_2_ 95% on room air). Baseline laboratory values are shown in Table 1. ECG showed sinus tachycardia with diffuse ST-segment elevation suggestive of acute pericarditis (Figure 2A). There were no acute processes on chest x-ray (Figure 2B). Day 1 TTE revealed normal ejection fraction (65%), moderate circumferential pericardial effusion, and exaggerated RV-LV interdependence with increased respirophasic variation in transmitral velocities (20-25%) but not meeting criteria for cardiac tamponade (Figure 2C). Vancomycin and meropenem were started for empiric antimicrobial coverage. Annulus reversus found on repeat TTE (higher mitral medial tissue velocity {E’} than lateral tissue velocity in patients with constrictive pericarditis, in contrast to normal subjects whose lateral mitral E’ is higher) led to the diagnosis of effusive-constrictive pericarditis on day three. Due to persistent leukocytosis despite broad-spectrum antimicrobial coverage, purulent pericarditis was suspected. Surgical pericardial drainage was performed on day three and resulted in drainage of 300 mL of purulent pericardial fluid (Table 3). A pericardial drain was placed for continued drainage. Blood cultures grew *Streptococcus pneumoniae* and antimicrobial therapy was switched to ceftriaxone based on culture and sensitivity data. There was continued drainage (40-100 mL/day) of pericardial fluid, but patient clinically deteriorated with worsening leukocytosis, eventually requiring non-invasive ventilatory support for impending respiratory failure. TTE on day six showed a small loculated pericardial effusion at the apex (Figure 2D). To improve pericardial drainage, r-tPA was infused through the drain. Patient was treated with three doses of 10 mg alteplase over three consecutive days beginning on day six, yielding 800 mL, 200 mL, and 180 mL pericardial fluid after each dose, respectively. Following this intervention, clinical status improved, and pericardial drain was removed on day 15. Patient remained hospitalized for an additional two weeks due to dorsal left-hand abscess requiring surgical drainage. Follow-up TTE on day 23 demonstrated resolution of previously noted annulus reversus and loculated apical pericardial effusion (Figure 2E). Patient was eventually discharged on an intravenous course of ceftriaxone to complete six weeks of antibiotic therapy.

## Discussion

In the pre-antibiotic era, purulent pericarditis was a frequent complication of pneumococcal pneumonia. It is usually seen in patients with nosocomial bloodstream infections, post-thoracic surgery, chest trauma, and immunosuppression as a consequence of direct spread from intrathoracic focus (most commonly *Streptococcus pneumoniae*), hematogenous spread (most commonly *Staphylococcus aureus* and various streptococci), or extension from a subdiaphragmatic source [1,4]. Mortality of patients with infective pericarditis appears to be lower in the modern era (2.3%) compared to older cohorts [3]. Pericardial drainage and antimicrobial coverage are the cornerstones of therapy. It can be achieved with either pericardiocentesis (echocardiography or fluoroscopy guided) or pericardial window. The former is a simpler and faster approach, but increased fluid viscosity and presence of loculations can impede complete drainage. Furthermore, pericardial constriction may occur following recovery if pericardiocentesis is the primary drainage method [5]. With the latter approach, digital lysis (manual breakdown by insertion of fingers during surgery) of adhesions and loculations can be accomplished. Combination of limited surgical drainage through a subxiphoid pericardial window with intrapericardial fibrinolysis could prevent the more invasive options, including wide drainage through full sternotomy or pericardiectomy [2]. Nevertheless, if pericardiocentesis is not feasible or if fibrinolysis fails, pericardiectomy is required to achieve full evacuation of the purulent effusion to control the source of infection.

tPA is a naturally occurring protein secreted primarily by vascular endothelial cells. It forms a tPA/fibrin unit that binds to plasminogen at the site of clot. The complex then converts plasminogen to plasmin inducing local fibrinolysis [6]. This process taking place in the pericardial space facilitates drainage and penetration of antibiotics into the pericardium by dissolution of loculations and areas of pericardial thickening resulting from pathological fibrin deposits [7]. There is limited literature on the utility of fibrinolytic therapy in purulent pericarditis. In a systematic review, Wiyeh et al. studied the efficacy and safety of intrapericardial fibrinolysis in pericardial effusion analyzing 17 case reports, 11 case series, and a randomized controlled trial (RCT), totaling 109 patients. The etiology was infectious in most patients, *Staphylococcus aureus *being the most common organism. Streptokinase or urokinase was mostly used, while only four patients received r-tPA. In 94 out of 109 participants (86%) treated with intrapericardial fibrinolysis, there were no adverse events. Fifteen patients developed complications which included pericardial constriction, persistent pericardial effusion, and cardiac tamponade. Hemorrhage was the most common short-term unfavorable outcome observed in 21 patients. As this review included mostly case reports and case series, which inherently carry a high risk for bias, efficacy and safety of intrapericardial fibrinolysis in preventing the complications of pericardial effusion could not be established. The RCT which was included also had an unclear or high risk of bias related to selection, blinding of participants, and outcome assessment [8]. Due to concerns about the safety of urokinase manufacturing, the product was withdrawn from the market in 1998. In 2005, streptokinase was taken off the US market due to concerns regarding allergic reactions [6].

Data on the use of r-tPA is limited to a few case reports. Multiple authors have used various agents at various doses [6,7,9,10]. In each of these cases, the trigger to initiate fibrinolysis was inadequate fluid drainage and/or lack of clinical improvement. Normal saline was used as the solvent for r-tPA in all instances. In each case, r-tPA led to enhanced drainage with clinical improvement. No adverse effects were noted except in the study by Johnson et al., where a transient episode of hemodynamically stable atrial fibrillation was noted - which was considered unrelated to r-tPA and likely due to irritation of the atrial wall by the catheter during injection [9]. Both of our patients had inadequate drainage from the pericardial drains, and r-tPA was used due to presence of loculations. Alteplase at three doses of 10 mg each, dissolved in normal saline, was infused into the pericardial space for three days. In case 1, the initial post-procedural TTE on day eight showed constrictive pericarditis physiology, however, these features were transient and were not seen on subsequent TTEs at four, 19, and 24 months. He appeared well on subsequent clinic visits, except for mild symptoms attributable to his other comorbidities. Case 2 did not have features of constrictive pericarditis on follow-up TTE. Our cases demonstrate role of r-tPA in safely improving pericardial drainage, thereby avoiding pericardiectomy. Use of r-tPA also has the potential to prevent future incidences of pericardial constriction, however, further long-term studies are needed in this regard before a definite conclusion can be made.

## Conclusions

r-tPA therapy may be beneficial in patients with purulent pericarditis, especially when initiated early after pericardial drain placement to facilitate pericardial drainage. It has the potential to prevent occurrence of pericardial constriction, however, further studies regarding long-term outcomes are needed. r-tPA therapy is relatively safe based on available data, especially when compared to older fibrinolytic agents which had significant adverse effects. A randomized controlled trial with large sample size of patients with purulent pericarditis is needed to assess the efficacy and long-term outcomes of intrapericardial r-tPA treatment.
